# Rapid and pervasive development- and tissue-specific homeolog expression partitioning in newly formed inter-subspecific rice segmental allotetraploids

**DOI:** 10.1186/s12864-018-5150-7

**Published:** 2018-10-19

**Authors:** Long Zhao, Lei Han, Chaoxia Xiao, Xiuyun Lin, Chunming Xu, Chunwu Yang

**Affiliations:** Key laboratory of Molecular Epigenetics of Ministry of Education (MOE), Northeast Normal University, Changchun, 130024 China

**Keywords:** Rice, Whole-genome duplication, Homeologous expression, Development, Tissue-specific silence

## Abstract

**Background:**

In diverse plant taxa, whole-genome duplication (WGD) events are major sources of phenotypic novelty. Studies of gene expression in synthetic polyploids have shown immediate expression and functional partitioning of duplicated genes among different tissues. Many studies of the tissue-specific homeolog expression partitioning have focused on allopolyploids that have very different parental genomes, while few studies have focused on autopolyploids or allopolyploids that have similar parental genomes.

**Results:**

In this study, we used a set of reciprocal F1 hybrids and synthetic tetraploids constructed from subspecies (japonica and indica) of Asian rice (*Oryza sativa* L.) as a model to gain insights into the expression partitioning of homeologs among tissues in a developmental context. We assayed the tissue-specific silencing (TSS) of the parental homeologs of 30 key genes in the hybrids and tetraploids relative to the in vitro “hybrids” (parental mixes) using Sequenom MassARRAY. We found that the parental mix and synthetic tetraploids had higher frequencies of homeolog TSS than the F1, revealing an instantaneous role of WGD on homeolog expression partitioning.

**Conclusions:**

Our observations contradicted those of previous studies in which newly formed allopolyploids had a low TSS frequency, similar to that of F1 hybrids, suggesting that the impact of WGD on homeolog expression requires a longer time to manifest. In addition, we found that the TSS frequency in the tetraploids varied at different growth stages and that roots had a much higher frequency of TSS than leaves, which indicated that developmental and metabolic traits may influence the expression states of duplicated genes in newly formed plant polyploids.

**Electronic supplementary material:**

The online version of this article (10.1186/s12864-018-5150-7) contains supplementary material, which is available to authorized users.

## Background

Polyploidy or whole-genome duplication (WGD) is a driving force in plant and vertebrate evolution, with all angiosperms having undergone WGD in their evolutionary histories [1–4]. Polyploidization occurred before the divergence of seed plants [3]. The common occurrence of WGD suggests an evolutionary advantage to plants having multiple genomes, resulting in polyploids having a greater adaptability than their diploid progenitors [5]. Polyploidy may have largely contributed to plant diversity because it provides the evolution of novel phenotypes with raw materials through the selection, retention and functional alteration of duplicate genes [6]. Duplicated genes caused by WGD may experience three fates: nonfunctionalization, neofunctionalization and subfunctionalization [7]. In newly formed polyploids, the division and alteration of ancestral patterns of duplicated genes are termed subfunctionalization (the two duplicates might divide the original function) and neofunctionalization (producing novel functions), respectively [6]. Nonfunctionalization is considered the complete silencing or loss in all cell types and development stages, or null mutation [7]. Following WGD, both subfunctionalization and neofunctionalization may be significant ways to retain both copies, which is a precursor of future evolution [8]. Although a much effort has gone into understanding the tissue-specific expression of duplicated genes in some plants [6, 8–12], only a few studies have focused on newly formed polyploids, such as cotton tetraploid [1, 6, 13] and *Tragopogon miscellus* [8, 11]. The tissue-specific expression pattern may be dependent on the particular genotypes and genes, the cellular context and environmental conditions. Therefore, the functional significance needs to be investigated in each case. To our knowledge, many studies of the tissue-specific expression have focused on allopolyploids that have very different parental genomes, such as cotton and *T. miscellus* [1, 6, 8, 9, 11–16], while limited studies have focused on autopolyploids or allopolyploids that have similar parental genomes. Although synthetic tetraploid rice shows extensive gene expression [17] and phenotypic alterations [18], the tissue- and development-specific expression of homeologs were not investigated.

Here, we used a model system involving the two Asian rice (*Oryza sativa* L.) subspecies, japonica and indica, which were both domesticated from a common wild progenitor, *Oryza rufipogon*, approximately 9,000 years ago [14]. The two subspecies were domesticated to generate extensive genetic divergences and to obtain distinct growth, developmental and agronomic traits [17, 19]. In this study, a japonica rice (cv. Nipponbare) and an indica rice (cv. 9311), their reciprocal F1 hybrids and synthetic tetraploids were employed as a study system. This is an excellent system to assay the evolution of new genes and the relationship between genomic shock and phenotype alteration in young polyploids because high-quality genome sequences for each subspecies can be accessed. In the present study, we randomly selected 30 genes of eight key pathways to gain insights into the expression partitioning of homeologs among tissues and during development. We observed rapid and pervasive tissue-specific silencing (TSS) of homeologs in newly formed rice tetraploids but not in F1 hybrids, indicating an instantaneous creative role for WGD in the genome alteration. Interestingly, our results were not consistent with previous studies in cotton [6] and *T. miscellus*, in which newly formed allopolyploids had a low TSS frequency similar to that of the F1 hybrid [11], and in which the partitioning of homeologs among the tissues rarely came from instantaneous effects of GWD but primarily came from the long-term effects of evolution and domestication.

## Methods

### Plant materials

Construction of plant materials were described in our previous work [17]. Reciprocal F1 hybrids N9 and 9 N were produced by crossing the two fully sequenced rice cultivars, Nipponbare and 9311. These two cultivars represent the two rice subspecies of Asian rice, japonica and indica, respectively. The tillers of the F1 hybrid plants were treated with colchicine to induce WGD. The synthetic rice allotetraploids were then self-pollinated for two generations under field conditions. All doubled plants with a confirmed euploid chromosome number were used for tissue collection [17]. All seeds used in this study were kindly provided by Dr. Bao Liu from Northeast Normal University (Changchun, China).We labeled cv. Nipponbare and cv. 9311 as ‘N’ and ‘9’, reciprocal hybrids as ‘N9’ and ‘9 N’, and reciprocal synthetic allotetraploids as ‘NN99’ and ‘99NN’. The maternal parents of 99NN/9 N and NN99/N9 are cv. 9311 and cv. Nipponbare, respectively. In this study, we randomly selected 10 ‘9’ plants, 10 ‘N’ plants, 7 ‘99NN’ plants, 7 ‘NN99’ plants, 4 ‘9 N’ plants and 2 ‘N9’ plants. The seeds were germinated and grown in Petri dishes for 6 d. The seedlings were then transferred to buckets containing 20 L of sterile nutrient solution for culturing. The nutrient solution was replaced every 2 d. The nutrient solution used in this work provides the components described by the International Rice Research Institute, and contained 1.44 mM NH_4_NO_3_, 0.32 mM NaH_2_PO_4_, 0.6 mM K_2_SO_4_, 1.0 mM CaCl_2_, 1.6 mM MgSO_4_, 0.072 mM Fe-EDTA, 0.2 mM Na_2_SiO_3_, 9.1 μM MnCl_2_, 0.154 μM ZnSO_4_, 0.156 μM CuSO_4_, 18.5 μM H_3_BO_3_ and 0.526 μM H_2_MoO_4_ at pH 5.3. All plants were grown under the same controlled conditions in an artificial climate room at Northeast Normal University (Changchun, China). The growth conditions were maintained at 26 °C day and 20 °C night under16 h light at ~ 400 μmol m^− 2^ s^− 1^ for 38 d. After the 38 d of 16 h light, the light length was changed to 12 h.

### RNA extraction and Sequenom MassARRAY assays

The plant samples were collected to extract total RNA at tillering and booting stages, and DNA only at the booting stage. At the tillering stage, mature leaf and root tissues were collected and labeled as “L1” and “R1”. To minimize the mechanical damage from sampling to plant growth, we only collected a small plant sample for each rice line. The sampling is unlikely to have affected the expression partitioning of duplicate genes at the booting stage because the booting stage biomass is several times that of the tillering stage. After sampling at the tillering stage, the plants were continuously grown in the artificial climate room. At the booting stage, these plants were sampled again, and the mature leaf, root, flag leaf and young spike of the plants were collected and labeled as “L2”, “R2”, “F” and “S”, respectively. DNA was extracted with a modified CTAB protocol. The total RNA was extracted using TRIzol reagent (Invitrogen). The RNA was treated with DNaseI (Invitrogen) and reverse-transcribed using SuperScriptTM RNase H-Reverse Transcriptase (Invitrogen).We estimated the qualities of RNA samples using RNA gel, and quantified the RNA concentrations with a NanoDrop machine (Thermo Scientific, USA). Then RNA samples of cv. 9311 and cv. Nipponbare were mixed in an equal RNA concentration. The cDNAs were synthesized from the equal RNA mixes of cv. 9311 and cv. Nipponbare to construct in vitro “hybrids” (labeled as MIX) [11]. The SNPs between parental homeologs had been identified in our previous works by using RNA sequencing data and IGV software [17, 18]. The Sequenom MassARRAY assays were conducted according to the method of Buggs et al., 2011 [11]. MassARRAY primers and probes for genome-specific expression were designed using the Sequenom MassARRAY platform [18]. cDNA solution of 1 μL (10 ng μL^− 1^) for each sample was used to perform PCR amplification and single-base primer extension reactions according to manufacturer’s specifications (Sequenom). All expression data recovered from the MassARRAY were first filtered on the basis of internal measures of assay quality. The MassARRAY primers and probe were continuously filtered on the basis of assays of cv. Nipponbare and cv. 9311 DNA samples were mixed in known ratios (1:3, 1:2, 1:1, 2:1 and 3:1) and used to test the genome-specificity of each gene primers and probe. All genes were required to display a strong correlation (R^2^ > 0.9) between the expected and observed N:9 DNA ratios [6]. When the majority of these genes gave an expected result, the assays were deemed to be accurate. By this method, 30 assays were found to be accurate (Additional file 1: Table S1). The statistical significance of TSS data was determined using the Mann–Whitney test and Wilcoxon-matched pair analysis in SPSS 13.0.

## Results

### Homeolog partitioning among tissues

**Fig. 1:**
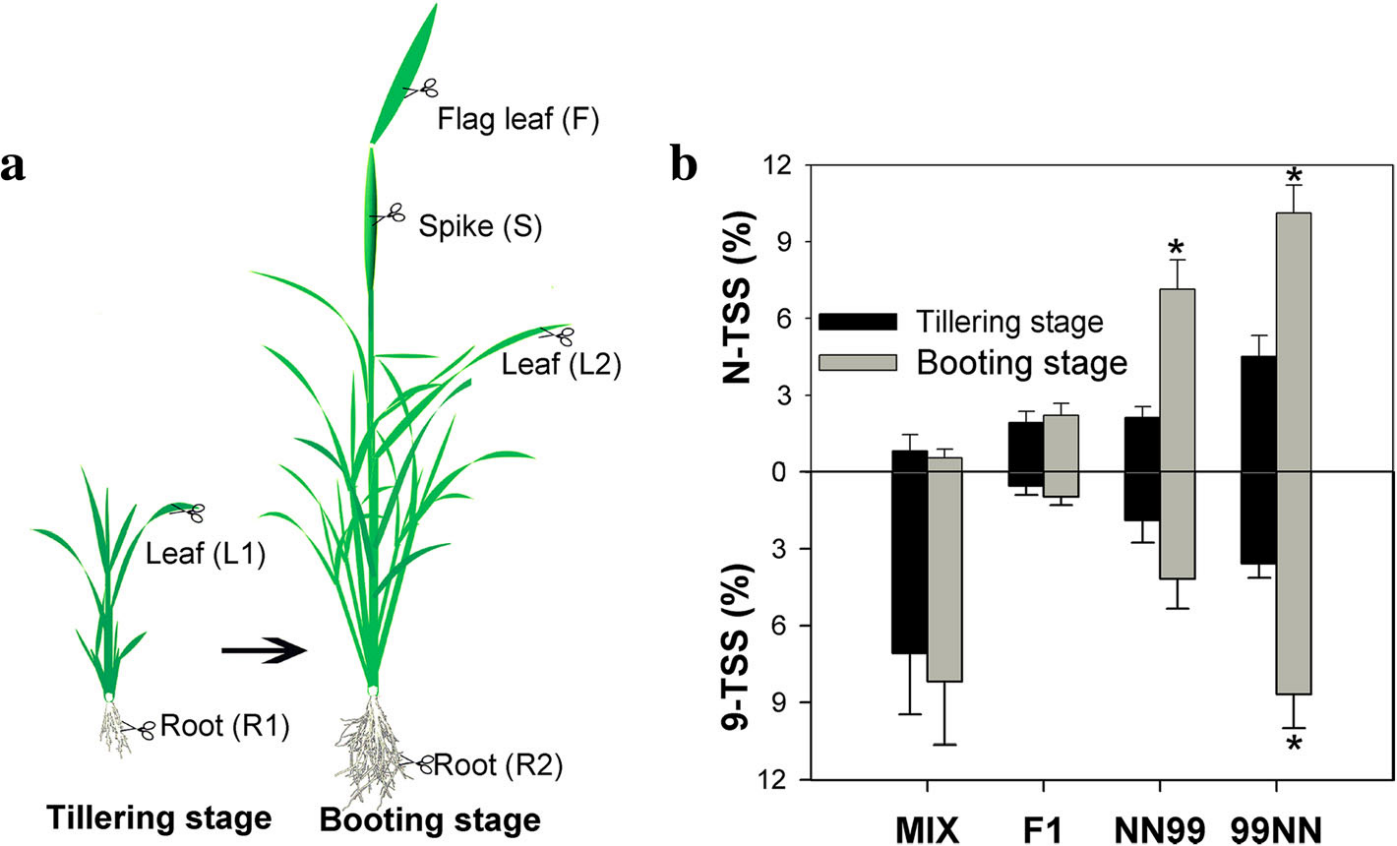
Expression partitioning of duplicate genes among tissues in newly synthetic rice allotetraploids. **a** Sampling method for different tissues and growth stages. Leaves and roots of each individual were sampled at the tillering stage, and then the sampled plants were still continuously cultivated through the booting stage. At the booting stage, the plants were sampled again, and a mature leaf, root, flag leaf and young spike were collected. L1, R1 represent expression of the mature leaf and root, respectively, at the tillering stage; L2, R2, F and S represent the expression of the mature leaf, root, flag leaf and young spike, respectively, at the booting stage. **b** Mean percentages of tissues showing tissue-specific silence (TSS) of alleles/homeologs for 30 genes at same growth stage (gene × tissue), in “*in vitro* hybrids” (2X, *n* = 6), F1 hybrids (2X, n = 6) and synthetic rice tetraploids (4X; NN99, *n* = 7 and 99NN, n = 7). Reciprocal synthetic tetraploids (99NN and NN99) were constructed from subspecies japonica (cv. Nipponbare, labelled as “N”) and indica (cv. 9311, labelled as “9”) rice. The maternal parents of 99NN and NN99 are cv. 9311 and cv. Nipponbare, respectively. The values are means of TSS (%) of six to seven biological replicates (individuals) ± standard error. * indicates a significant difference between both stages within a rice line, according to a Mann–Whitney test (*P* < 0.05)

**Fig. 2:**
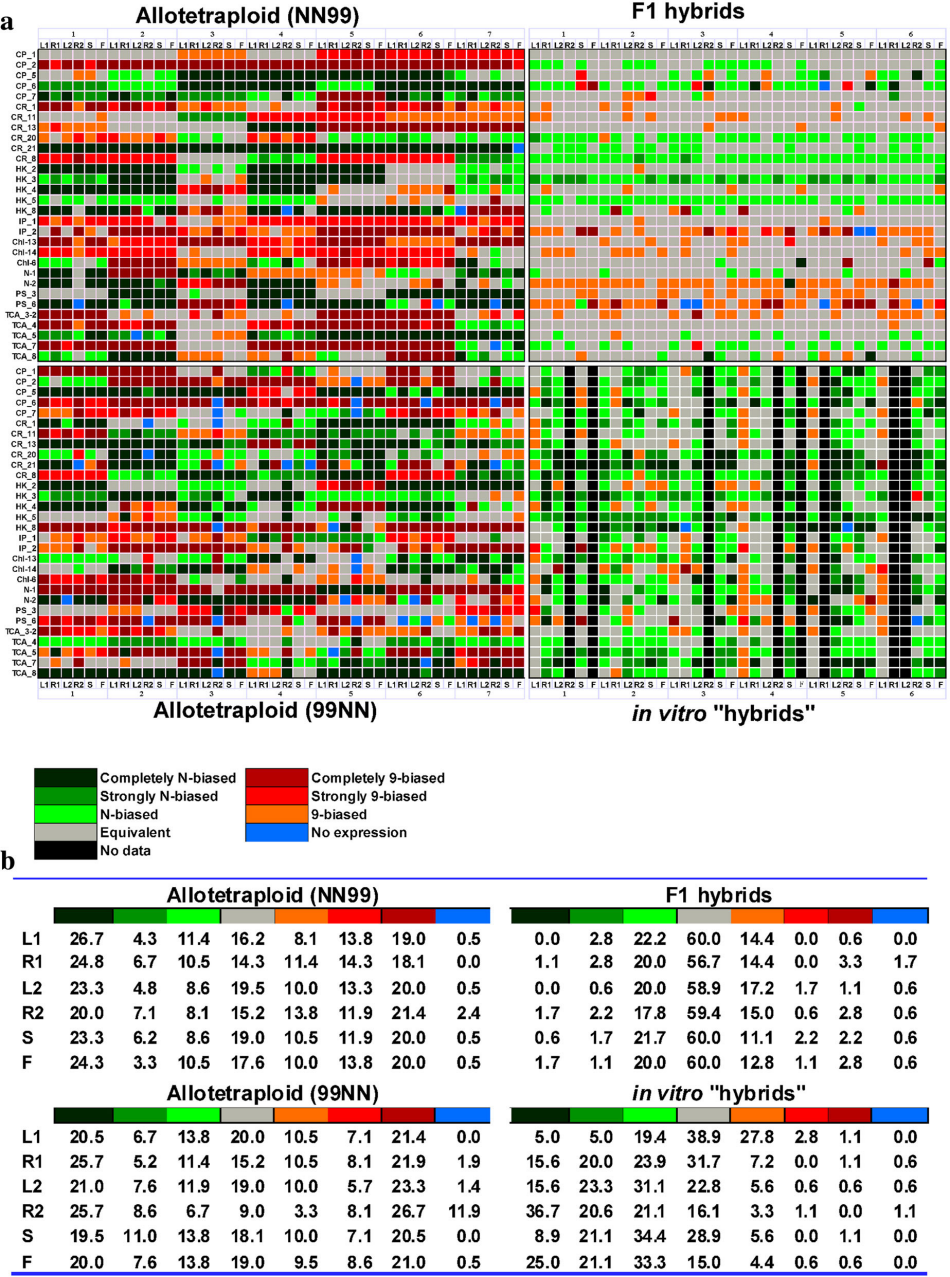
Expression partitioning of duplicate genes in newly synthetic allotetraploid rice. **a** Tissue-specific relative expression levels of gene copies derived from japonica (cv. Nipponbare, labelled as “N”) and indica (cv. 9311, labelled as “9”) rice in in vitro “hybrids (2X),” F1 hybrids (2X) and reciprocal synthetic allotetraploids (S3 generation, NN99 and 99NN). Reciprocal hybrids and allotetraploids were constructed from japonica and indica rice subspecies. 99NN and NN99 had cv. 9311 and cv. Nipponbare as maternal parents, respectively. In all four plant groups, the biased expression categories corresponded to the percentage contribution of japonica rice homeolog (N) to total transcripts [N/(9 + N) × 100%]: completely N-biased (95–100% N expression, 9-silence); strongly N-biased (80–95% N expression); N-biased (60–80% N expression); equivalent (40–60% N expression); completely 9-biased (0–5% N expression, N-silenced); strongly 9-biased (5–20% N expression); 9-biased (20–40% N expression). Silencing of both copies was considered as “No expression”. **b** Distribution of homeolog-specific expression states within different tissue types and among plant groups. L1, R1 represent expression of the mature leaf and root, respectively, at the tillering stage; L2, R2, F and S represent the expression of the mature leaf, root, flag leaf and young spike, respectively, at the booting stage

We labeled cv. Nipponbare and cv. 9311 as ‘N’ and ‘9’, reciprocal hybrids as ‘N9’ and ‘9 N’, and reciprocal synthetic allotetraploids as ‘NN99’ and ‘99NN’. The maternal parents of 99NN/9 N and NN99/N9 are cv. 9311 and cv. Nipponbare, respectively. In F1 and MIX, ‘N’ and ‘9’ represent cv. Nipponbare allele and cv. 9311 allele, respectively**.** In the allotetraploids, ‘N’ and ‘9’ represent cv. Nipponbare homeolog and cv. 9311 homeolog, respectively. In this study, we randomly chose 30 genes of eight key pathways to investigate the expression partitioning of homeologs among tissues and during development. These pathways include circadian and photomorphogenesis, chromosome regulator, housekeeping, ion transportation pathway, chlorophyll metabolism, photosynthesis, nitrogen metabolism and tricarboxylic acid cycle (Additional file 1: Table S1). The 30 genes all play key roles in their corresponding pathways. To accurately calculate the frequency of TSS during development, we carried out a specific sampling method: the same individual was sampled twice at both tillering and at booting stages (Fig. 1a). This special sampling method can minimize “the effects of individual to individual” caused by variation in the genomic background during the assessment of the TSS of homeologs during development. We analyzed the biased expression of homeologs in three plant groups: “*in vitro* hybrids”(MIX), F1 hybrids (9 N and N9), and synthetic rice tetraploids (NN99 and 99NN), using Sequenom MassARRAY (Fig. 2 and Additional file 2: Figure S1, Additional file 3: Figure S2, Additional file 4: Figure S3, Additional file 5: Figure S4, Additional file 6: Figure S5, Additional file 7: Figure S6, Additional file 8: Figure S7, Additional file 9: Figure S8). MIX had a higher percentage of TSS than F1 (Figs. 1 and 2), revealing that hybridization had caused an immediate and saltational disruption of parental expression patterns. In MIX, 74% of locus (gene×individual) showed unequal expression pattern of ‘9’ allele and ‘N’ allele, indicating that there are a large number of gene expression differences between cv. 9311(‘9’) and cv. Nipponbare (‘N’). In contrast to MIX, F1 has lower percentages of silencing event and TSS (Fig. 2 and Additional file 3: Figure S2, Additional file 4: Figure S3, Additional file 5: Figure S4, Additional file 6: Figure S5, Additional file 7: Figure S6, Additional file 8: Figure S7, Additional file 9**:** Figure S8), and 60% of locus of the F1 plants displayed equal expression pattern of ‘9’ allele and ‘N’ allele.

Next, we examined the effects of WGD events on the expression of duplicate genes through comparing F1 and tetraploids (99NN and NN99). We calculated the percentages of TSS and the percentages of biased expression between ‘9’ copy and ‘N’ copy. We determined the percentage of TSS for homeologs between L1 and R1 at the tillering stage, as well as TSS among L2, R2, F and S at the booting stage (Fig. 1b and Additional file 10: Table S2). We also considered the percentage of TSS among all six tissues measured (Additional file 2: Figure S1 and Additional file 10: Table S2). Both NN99 and 99NN had higher frequencies of TSS and the biased expression than the those of F1, with 99NN > NN99.

### Effects of development on expression partitioning of homeologs

**Fig. 3:**
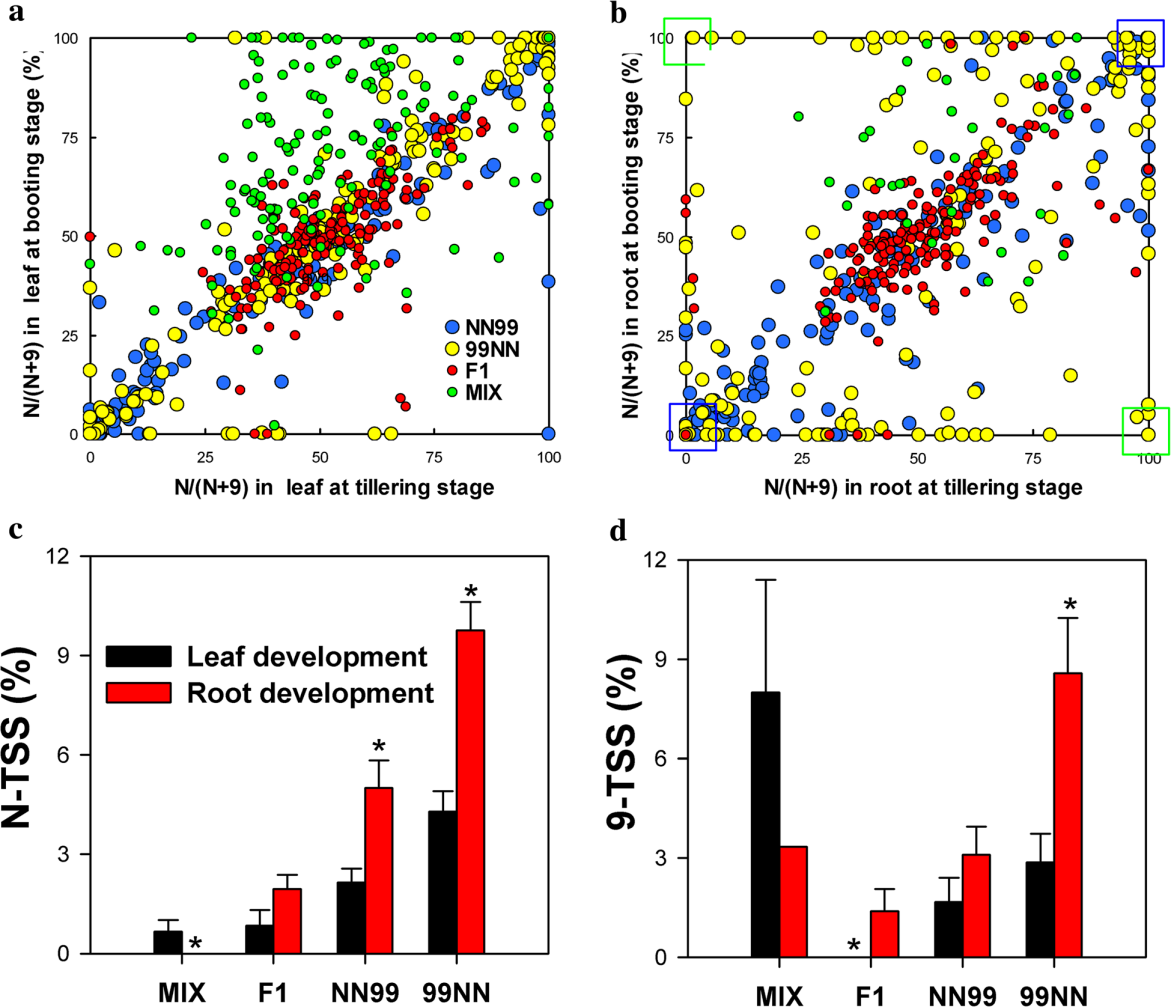
Effect of development on expression partitioning of duplicate genes in in vitro “hybrids” (MIX), F1 hybrids (F1) and reciprocal synthetic allotetraploids (S3 generation, NN99 and 99NN). **a**–**b** Relative homeolog expression was expressed as the percentage contribution of japonica rice homeolog (N) to total transcripts [N/ (9 + N) × 100%]. **b** blue squares represent tissues that lost some homeologs (nonfunctionalization), green squares represent reciprocal tissue-specific silencing (TSS) caused by development. In the two panels, the icons that aggregated to the *x*-axis, *y*-axis or external frame lines indicated that the genes underwent TSS. **c**–**d** Mean percentages of tissues showing TSS of alleles/homeologs caused by development (across the two stages) within same tissue types (roots or mature leaves). Figure c and Figure d show mean TSS (%) values for the japonica (N) and indica (9) subgenome-copies, respectively. The values are means of six to seven biological replicates (individuals) ± standard error. * indicates a significant difference between root and leaf within a rice line, according to a Mann–Whitney test (*P* < 0.05)

To assay the effects of development on homeolog expression partitioning, we compared homeologous expression patterns of the same organ (leaf or root) at two growth stages (Fig. 3). To find the TSS only caused by development, we calculated TSS between L1 and L2 that were sampled at tillering and booting stages, respectively, as well as TSS between R1 and R2 (Fig. 3c and d). The TSS was perceived to be caused by developmental processes (Fig. 3c and d). In Fig. 3a and b, the icons aggregated to a diagonal line, indicating that development did not produce any effects on homeologous expression partitioning, and the icons aggregated to the *x*-axis, *y*-axis or external frame lines indicated the genes with TSS. Most icons representing F1 hybrids aggregated to the diagonal line, and most icons representing 99NN and NN99 leaves were also close to the diagonal line. However, in 99NN and NN99, most icons representing roots were far from the diagonal line. Thus, the developmental processes only produced a small effect on the homeologous expression partitioning of F1 plants, whereas they strongly influenced the homeologous partitioning of NN99 and 99NN (Fig. 3a and b). The TSS frequency caused by development was much higher in roots than in leaves (Fig. 3c and d).

## Discussion

### Both WGD and hybridization instantaneously induced the pervasive alteration of homeolog partitioning among tissues

The processes of selection and retention of duplicate genes (diploidization) after a single-gene duplication or WGD are important driving forces for the phenotypic evolution of plants [16]. Several studies have made a connection between homeologous partitioning and phenotypic innovation. In Flaveria plants, the functional partitioning following the duplication of two key C4 photosynthetic genes, carbonic anhydrase [20] and phosphoenolpyruvate carboxylase [21], immediately led to a shift in the photosynthetic pathway from C3 to C4. In tomato, the duplication of the *QD12* gene created a new copy (*SUN* gene) through a copia-like retrotransposon. The new *SUN* copy was highly expressed in tomato fruit and was important for the elongated fruit shape [22]. Thus, homeologous partitioning following the duplication of a single gene can generate phenotypic innovation. In many newly formed polyploids, WGD can induce massive amounts of expression and functional alterations of homeologs, which may rapidly promote the formation of new species or new traits [2, 22, 23]^.^ In this study, we randomly chose 30 genes of eight key pathways to investigate the expression partitioning of homeologs among tissues and during the development.

Firstly, we surveyed the effects of hybridization events (first step of polyploidization) on the expression of alleles by comparing MIX and F1. MIX had a higher percentage of TSS than F1 (Figs. 1 and 2). In MIX, 74% of locus (gene×individual) showed unequal expression pattern of ‘9’ allele and ‘N’ allele (Fig. 2). In contrast, F1 plants have lower percentages of silencing event and TSS than MIX (Fig. 2 and Additional file 3: Figure S2, Additional file 4: Figure S3, Additional file 5: Figure S4, Additional file 6: Figure S5, Additional file 7: Figure S6, Additional file 8: Figure S7, Additional file 9: Figure S8), and 60% of the locus of the F1 plants displayed equal expression pattern of ‘9’ allele and ‘N’ allele, revealing that hybridization had caused an immediate and saltational disruption of parental expression patterns, and the original gene expression regulation was widely relaxed. In fact, in MIX, unequal expression pattern of ‘9’ allele and ‘N’ allele represented gene expression difference between cv. 9311(‘9’) and cv. Nipponbare (‘N’). In natural condition (MIX), for some genes, differences between cv. 9311(‘9’) and cv. Nipponbare (‘N’) in allele expression may be attributable to their different *Trans* regulators; however, following genomic merger (hybridization), the *Trans* regulators of the local subgenome may mediate the expression of the allele of another foreign subgenome. In F1 rice plants, for some genes, given that promoter sequences of ‘9’ allele and ‘N’ allele have a relatively few SNP or not SNP, their *Trans* regulators may have equal frequency to bind their CIS sequences and will result in such equal expression of ‘9’ allele and ‘N’ allele. Homeolog partitioning of FtsH protease gene (HK-5) may be a typical support for this hypothesis. ‘9’ allele of this gene was completely silenced in both root tissues (R1 and R2) of all MIX plants, but the expression of the ‘9’ allele was re-activated in R1 and R2 of all F1 plants and all NN99 plants (Additional file 3: Figure S2). Expression of ‘9’ allele of HK-5 gene in the F1 hybrids and NN99 plants may be activated by the *Trans* regulators of ‘N’ allele of HK-5 gene. Interestingly, in R1 and R2 of some 99NN plants, the expression of the ‘9’ copy was silenced again and was restored to its original expression pattern of MIX (Additional file 3: Figure S2). The re-silencing may be mediated by many complex mechanisms such as small interfering RNA, DNA methylation, transposon, and retrotransposon, which should be investigated in the future.

Next, we examined the effect of WGD events on the expression of duplicate genes by comparing F1 and tetraploids (99NN and NN99). We found that both NN99 and 99NN had higher frequencies of TSS and biased expression than those of F1, with 99NN > NN99 (Figs. 1 and 2 and Additional file 2: Figure S1). This indicated that, in rice tetraploids, WGD instantaneously affected the genetic consequences of hybridization events and induced the rapid and pervasive alteration of tissue-specific expression (Figs. 1, 2, and 3). For instance, in all tissues of all F1 plants, response regulator receiver domain-containing protein (CP-2) gene showed equal expression of ‘9’ allele and ‘N’ allele, whereas ‘N’ homeolog of this gene was silenced in most tissues of all NN99 plants and two 99NN plants (Additional file 3: Figure S2). This donated that WGD may instantaneously shock the genetic consequences of hybridization in CP-2 gene, which potentially influenced the phenotypes of the newly formed rice tetraploids because CP-2 gene is a key gene for circadian and photomorphogenesis. Our results contradict those of the studies in cotton [6] and *T. miscellus* [11], in which newly formed allopolyploids had low TSS frequencies similar to those of the respective F1 hybrids. We propose that, in these two species, the impact of WGD on homeologous TSS needs a longer time to manifest. This difference may be rooted in their distinct heterozygosities. The subgenomes of cotton and *T. miscellus* synthetic allopolyploids come from different species, and the subgenomes in rice tetraploids, being subspecies, are more closely related.

### Development is instrumental in expression partitioning of homeologs

In newly formed rice tetraploids, the most common fates for duplicate genes are that one of the copies retains the ancestral level of function and expression, and the second copy is lost (Fig. 2). In 99NN and NN99, 14 cases of reciprocal TSS (subfunctionalization) were found. Both copies of duplicate genes with subfunctionalization (reciprocal TSS) may be permanently preserved in subsequent long-term evolution. In yeast, after a WGD event, both copies of duplicated genes are permanently preserved either by neofunctionalization or classical subfunctionalization [24]. This hypothesis was also supported by work on the synthetic cotton tetraploid, in which four genes showed similar silencing and/or expressional biases between synthetic and natural tetraploids, suggesting an evolutionary perseverance [13]. In an important review on the evolution of duplicated genes, it has been pointed out that the retention of duplicated genes after WGD events affects the evolution of plants [16]. Some key questions were still unanswered, including: which duplicated genes are retained? and which factors influence the behavior of duplicated genes? Our results might be helpful in answering these questions [16]. We wondered whether development is instrumental in the expression partitioning of homeologs and whether different organs differ in the homeologous partitioning styles during development.

Our results displayed that the developmental processes only produced a small effect on the homeologous expression partitioning of the F1 plants, whereas they strongly influenced the homeologous partitioning of NN99 and 99NN (Figs. 3a and b). For instance, in CP_1 gene of 99NN plant 8_4 (Additional file 4: Figure S3), CR_1 gene of 99NN plant 8_5 (Additional file 4: Figure S3), Chl_6 gene of NN99 plant 7_9 (Additional file 8: Figure S7) and HK_5 gene of NN99 plant 7_9 (Additional file 3: Figure S2), the developmental processes led to homeologous expression partitioning in these rice tetraploid plants but not in the F1 plants. The homeologous expression partitioning may be mediated by different mechanisms, such as micro-RNA, DNA methylation, transposon insertion and histone modification. It also is likely that the developmental processes led to homeologous function partitioning in these genes, which should be further investigated through CRISPR/Cas9 or other knockout transgenic and over-expression experiments. In addition, the TSS frequency caused by development was much higher in roots than in leaves (Figs. 3c and d). When crossing the tillering and booting stages, leaf tissue had much smaller metabolic changes than root tissue. At the booting stage, the vegetative growth rate of rice is lowered and reproductive development begins. The root activity starts to decline and root tissues may experience large metabolic shifts. This developmental process may affect the expression of homeologs. Thus, we propose that both developmental and metabolic traits influence the expression states of duplicate genes in newly formed polyploids.

## Conclusions

The F1 plants showed lower percentages of homeologous TSS than MIX, revealing that hybridization had caused an immediate and saltational disruption of parental expression patterns, and the original gene expression regulation was widely relaxed. The synthetic rice tetraploids had higher frequencies of homeologous TSS than the F1, displaying an instantaneous role of WGD on homeolog expression partitioning. Additionally, the TSS frequency in the rice tetraploids varied at different growth stages and the roots had a much higher frequency of TSS than the leaves. This indicated that developmental process and metabolic traits may influence the expression states of duplicated genes in newly formed rice polyploids.

## Additional files

Additional file 1: **Table S1.** Genes used in Sequenom MassARRAY assays. (XLS 42 kb)

Additional file 2: **Figure S1.** Mean percentage of tissues showing tissue-specific silencing (TSS) of alleles/homeologs in all six tissues (gene × tissue), and in in vitro “hybrids (2X, n = 6)”, F1 hybrids (2X, *n* = 6), and synthetic rice tetraploids (NN99 and 99NN, *n* = 7). The values are means of six to seven biological replicates (individuals) ± standard error. (a) Means followed by different letters are significantly different according to a Mann–Whitney test (*P* < 0.05). (b) Wilcoxon-matched pair test among four plant groups for total TSS (30 genes). (TIF 106 kb)

Additional file 3: **Figure S2.** Tissue- and homeolog-specific gene expression levels among four gene pairs in in vitro “hybrids” (2X), F1 hybrids (2X) and synthetic allotetraploids (S3). Tissues are represented along the *x*-axis, while relative homeolog expression [N/ (9 + N) × 100%] is represented on the *y*-axis. Relative homeolog expression was expressed as the percentage contribution of japonica rice homeolog (N) to total transcripts [N/ (9 + N) × 100%]. In in vitro “hybrids”, ●, ○, ▼, △, ■ and □ represent L2, L3, L5, L6, L7 and L8 plants, respectively; In F1 hybrids, ●, ○, ▼, △, ■ and □ represent A1, A2, A3, A7, B1, and B2 plants, respectively; In synthetic allotetraploids (NN99), ●, ○, ▼, △, ■, □ and ◆ represent 7_1, 7_3, 7_4, 7_5, 7_6, 7_8 and 7_9 plants, respectively. In synthetic allotetraploids (99NN), ●, ○, ▼, △, ■, □ and ◆ represent 8_1, 8_3, 8_4, 8_5, 8_6, 8_7 and 8_9 plants, respectively. CP, Circadian and Photomorphogenesis; CP-2, response regulator receiver domain-containing protein (PRR9); CP-6, FKF1; CR, Chromosome R, CR11–1: histone H1; HK-Housekeeping gene, HK-5: FtsH protease. L1, R1 represent expression of the mature leaf and root, respectively, at the tillering stage; L2, R2, F and S represent the expression of the mature leaf, root, flag leaf and young spike, respectively, at the booting stage. (TIF 252 kb)

Additional file 4: **Figure S3.** Tissue-specific and homeolog-specific gene expression in in vitro “hybrids” (2X), F1 hybrids (2X), and synthetic allotetraploids (S3). Reciprocal hybrids and allotetraploids. Tissues are represented along the *x*-axis, while relative homeolog expression [N/ (9 + N) × 100%] is represented on the *y*-axis. Relative homeolog expression was expressed as the percentage contribution of japonica rice homeolog (N) to total transcripts [N/ (9 + N) × 100%]. In in vitro “hybrids”, ●, ○, ▼, △, ■ and □ represent L2, L3, L5, L6, L7 and L8 plants, respectively; In F1 hybrids, ●, ○, ▼, △, ■ and □ represent A1, A2, A3, A7, B1, and B2 plants, respectively; In synthetic allotetraploids (NN99), ●, ○, ▼, △, ■, □ and ◆ represent 7_1, 7_3, 7_4, 7_5, 7_6, 7_8 and 7_9 plants, respectively. In synthetic allotetraploids (99NN), ●, ○, ▼, △, ■, □ and ◆ represent 8_1, 8_3, 8_4, 8_5, 8_6, 8_7 and 8_9 plants, respectively. CP, Circadian and Photomorphogenesis; CP-1: COC1 gene; CP-5: TOC1, response regulator receiver domain containing protein; CP-7: LUX, MYB family transcription factor; CR, Chromosome R; CR-1: HTR707 | *Oryza sativa ssp. japonica* | transcript sequence | Histone H3 Family; L1, R1 represent expression of the mature leaf and root, respectively, at the tillering stage; L2, R2, F and S represent the expression of the mature leaf, root, flag leaf and young spike, respectively, at the booting stage. (TIF 263 kb)

Additional file 5: **Figure S4.** Tissue-specific and homeolog-specific gene expression in in vitro “hybrids” (2X), F1 hybrids (2X), and synthetic allotetraploids (S3). Reciprocal hybrids and allotetraploids. Tissues are represented along the *x*-axis, while relative homeolog expression [N/ (9 + N) × 100%] is represented on the *y*-axis. Relative homeolog expression was expressed as the percentage contribution of japonica rice homeolog (N) to total transcripts [N/ (9 + N) × 100%]. In in vitro “hybrids”, ●, ○, ▼, △, ■ and □ represent L2, L3, L5, L6, L7 and L8 plants, respectively; In F1 hybrids, ●, ○, ▼, △, ■ and □ represent A1, A2, A3, A7, B1, and B2 plants, respectively; In synthetic allotetraploids (NN99), ●, ○, ▼, △, ■, □ and ◆ represent 7_1, 7_3, 7_4, 7_5, 7_6, 7_8 and 7_9 plants, respectively. In synthetic allotetraploids (99NN), ●, ○, ▼, △, ■, □ and ◆ represent 8_1, 8_3, 8_4, 8_5, 8_6, 8_7 and 8_9 plants, respectively. CR, Chromosome R; CR-13: HUPB701, ubiquitin-conjugating enzyme; CR-20: SGS701, leafbladeless1; CR-21: NFF701, Nucleosome/chromatin assembly complex proteins (Cac1 homologs); CR-8: HTA706, Core histone H2A/H2B/H3/H4 domain containing protein; L1, R1 represent expression of the mature leaf and root, respectively, at the tillering stage; L2, R2, F and S represent the expression of the mature leaf, root, flag leaf and young spike, respectively, at the booting stage. (TIF 251 kb)

Additional file 6: **Figure S5.** Tissue-specific and homeolog-specific gene expression in in vitro “hybrids” (2X), F1 hybrids (2X), and synthetic allotetraploids (S3). Reciprocal hybrids and allotetraploids. Tissues are represented along the *x*-axis, while relative homeolog expression [N/ (9 + N) × 100%] is represented on the *y*-axis. Relative homeolog expression was expressed as the percentage contribution of japonica rice homeolog (N) to total transcripts [N/ (9 + N) × 100%]. In in vitro “hybrids”, ●, ○, ▼, △, ■ and □ represent L2, L3, L5, L6, L7 and L8 plants, respectively; In F1 hybrids, ●, ○, ▼, △, ■ and □ represent A1, A2, A3, A7, B1, and B2 plants, respectively; In synthetic allotetraploids (NN99), ●, ○, ▼, △, ■, □ and ◆ represent 7_1, 7_3, 7_4, 7_5, 7_6, 7_8 and 7_9 plants, respectively. In synthetic allotetraploids (99NN), ●, ○, ▼, △, ■, □ and ◆ represent 8_1, 8_3, 8_4, 8_5, 8_6, 8_7 and 8_9 plants, respectively. HK: Housekeeping gene; HK-2: Alpha tubulin; HK-3: Ubiquitin; HK-4: EF1d; HK-8-2: endo-1, 4-beta-glucanase; L1, R1 represent expression of the mature leaf and root, respectively, at the tillering stage; L2, R2, F and S represent the expression of the mature leaf, root, flag leaf and young spike, respectively, at the booting stage. (TIF 262 kb)

Additional file 7: **Figure S6.** Tissue-specific and homeolog-specific gene expression in in vitro “hybrids” (2X), F1 hybrids (2X), and synthetic allotetraploids (S3). Reciprocal hybrids and allotetraploids. Tissues are represented along the *x*-axis, while relative homeolog expression [N/ (9 + N) × 100%] is represented on the *y*-axis. Relative homeolog expression was expressed as the percentage contribution of japonica rice homeolog (N) to total transcripts [N/ (9 + N) × 100%]. In in vitro “hybrids”, ●, ○, ▼, △, ■ and □ represent L2, L3, L5, L6, L7 and L8 plants, respectively; In F1 hybrids, ●, ○, ▼, △, ■ and □ represent A1, A2, A3, A7, B1, and B2 plants, respectively; In synthetic allotetraploids (NN99), ●, ○, ▼, △, ■, □ and ◆ represent 7_1, 7_3, 7_4, 7_5, 7_6, 7_8 and 7_9 plants, respectively. In synthetic allotetraploids (99NN), ●, ○, ▼, △, ■, □ and ◆ represent 8_1, 8_3, 8_4, 8_5, 8_6, 8_7 and 8_9 plants, respectively. IP: Ion transportation Pathway; IP-1; HKT1;1-Na^+^ transporter; IP-2: Potassium channel AKT1; Chl: Metabolism chlorophyll; Chl-13: oxidoreductase, short chain dehydrogenase/reductase family domain containing protein; Chl-14: oxidoreductase, short chain dehydrogenase/reductase family domain containing family; L1, R1 represent expression of the mature leaf and root, respectively, at the tillering stage; L2, R2, F and S represent the expression of the mature leaf, root, flag leaf and young spike, respectively, at the booting stage. (TIF 278 kb)

Additional file 8: **Figure S7.** Tissue-specific and homeolog-specific gene expression in in vitro “hybrids” (2X), F1 hybrids (2X), and synthetic allotetraploids (S3). Reciprocal hybrids and allotetraploids. Tissues are represented along the *x*-axis, while relative homeolog expression [N/ (9 + N) × 100%] is represented on the *y*-axis. Relative homeolog expression was expressed as the percentage contribution of japonica rice homeolog (N) to total transcripts [N/ (9 + N) × 100%]. In in vitro “hybrids”, ●, ○, ▼, △, ■ and □ represent L2, L3, L5, L6, L7 and L8 plants, respectively; In F1 hybrids, ●, ○, ▼, △, ■ and □ represent A1, A2, A3, A7, B1, and B2 plants, respectively; In synthetic allotetraploids (NN99), ●, ○, ▼, △, ■, □ and ◆ represent 7_1, 7_3, 7_4, 7_5, 7_6, 7_8 and 7_9 plants, respectively. In synthetic allotetraploids (99NN), ●, ○, ▼, △, ■, □ and ◆ represent 8_1, 8_3, 8_4, 8_5, 8_6, 8_7 and 8_9 plants, respectively. Chl: Metabolism chlorophyll; Chl-6: uroporphyrinogen decarboxylase; N: Nitrogen metabolism; N-1: glutamate dehydrogenase protein; N-2: ferredoxin-dependent glutamate synthase precursor; PS: Photosynthesis; PS-3: glyceraldehyde-3-phosphate dehydrogenase; L1, R1 represent expression of the mature leaf and root, respectively, at the tillering stage; L2, R2, F and S represent the expression of the mature leaf, root, flag leaf and young spike, respectively, at the booting stage. (TIF 270 kb)

Additional file 9: **Figure S8.** Tissue-specific and homeolog-specific gene expression in in vitro “hybrids” (2X), F1 hybrids (2X), and synthetic allotetraploids (S3). Reciprocal hybrids and allotetraploids. Tissues are represented along the *x*-axis, while relative homeolog expression [N/ (9 + N) × 100%] is represented on the *y*-axis. Relative homeolog expression was expressed as the percentage contribution of japonica rice homeolog (N) to total transcripts [N/ (9 + N) × 100%]. In in vitro “hybrids”, ●, ○, ▼, △, ■ and □ represent L2, L3, L5, L6, L7 and L8 plants, respectively; In F1 hybrids, ●, ○, ▼, △, ■ and □ represent A1, A2, A3, A7, B1, and B2 plants, respectively; In synthetic allotetraploids (NN99), ●, ○, ▼, △, ■, □ and ◆ represent 7_1, 7_3, 7_4, 7_5, 7_6, 7_8 and 7_9 plants, respectively. In synthetic allotetraploids (99NN), ●, ○, ▼, △, ■, □ and ◆ represent 8_1, 8_3, 8_4, 8_5, 8_6, 8_7 and 8_9 plants, respectively. PS: Photosynthesis; PS-6: F-type H + −transporting ATPase subunit gamma; TCA: Kreb’s cycle; TCA3–2: Malate synthase-like family protein-1; TCA-4: NAD-dependent malic enzyme, mitochondrial precursor-1; TCA-7: NADP-dependent malic enzyme-2; TCA-8: Aconitate hydratase protein-1; TCA-5: NAD-dependent malic enzyme, mitochondrial precursor-2; L1, R1 represent expression of the mature leaf and root, respectively, at the tillering stage; L2, R2, F and S represent the expression of the mature leaf, root, flag leaf and young spike, respectively, at the booting stage. (TIF 370 kb)

Additional file 10: **Table S2.** Tissue-specific silencing (a) and development-specific silencing (b) at different growth stages. Mean percentages of tissues showing tissue-specific silence of alleles/homeologs at different stages. (XLS 41 kb)
